# Lessons from the Pacific programme to eliminate lymphatic filariasis: a case study of 5 countries

**DOI:** 10.1186/1471-2334-9-92

**Published:** 2009-06-12

**Authors:** Clare Huppatz, Corinne Capuano, Kevin Palmer, Paul M Kelly, David N Durrheim

**Affiliations:** 1 Hunter New England Population Health Unit, New South Wales Health, Locked Bag 10, Wallsend, Australia; 2 National Centre for Epidemiology and Population Health, College of Medicine, Biology and Environment, Australian National University, Canberra, Australia; 3 Pacific Programme to Eliminate Lymphatic Filariasis, Office of the World Health Organization Representative in the South Pacific, Suva, Fiji; 4 Country Office, World Health Organization, Apia, Samoa; 5 Hunter Medical Research Institute and School of Public Health and Medical Practice, University of Newcastle, Newcastle, New South Wales, Australia

## Abstract

**Background:**

Lymphatic Filariasis (LF) is an important Neglected Tropical Disease, being a major cause of disability worldwide. The Global Programme to Eliminate Lymphatic Filariasis aims to eliminate LF as a public health problem by the year 2020, primarily through repeated Mass Drug Administration (MDA). The Pacific region programme commenced in 1999. By June 2007, five of the eleven countries classified as endemic had completed five MDA campaigns and post-MDA prevalence surveys to assess their progress. We review available programme data and discuss their implications for other LF elimination programs in developing countries.

**Methods:**

Reported MDA coverage and results from initial surveys and post-MDA surveys of LF using the immunochromatographic test (ICT) from these five Pacific Island countries (Tonga, Niue, Vanuatu, Samoa and Cook Islands) were analysed to provide an understanding of their quality and programme progress towards LF elimination. Denominator data reported by each country programme for 2001 was compared to official sources to assess the accuracy of MDA coverage data.

**Results:**

Initial survey results from these five countries revealed an ICT prevalence of between 2.7 and 8.6 percent in individuals tested prior to commencement of the programme. Country MDA coverage results varied depending on the source of denominator data. Of the five countries in this case study, three countries (Tonga, Niue and Vanuatu) reached the target prevalence of <1% antigenaemia following five rounds of MDA. However, endpoint data could not be reliably compared to baseline data as survey methodology varied.

**Conclusion:**

Accurate and representative baseline and post-campaign prevalence data is crucial for determining program effectiveness and the factors contributing to effectiveness. This is emphasised by the findings of this case study. While three of the five Pacific countries reported achieving the target prevalence of <1% antigenaemia, limitations in the data preclude identification of key determinants of this achievement.

## Background

Lymphatic filariasis (LF), an infection caused by a mosquito borne parasite, is the second leading cause of disability worldwide, affecting more than 120 million people in 80 countries [1-3]. It is a major cause of physical and emotional suffering, as well as economic loss [4,5]. The three species of nematode worm that cause LF are *Wuchereria bancrofti*, *Brugia malayi *and *Brugia timori *[6]. Bancroftian filariasis accounts for 90% of cases worldwide [6], including all cases of LF in the Pacific [2].

In 1997 the World Health Organization (WHO) declared LF one of six potentially eradicable diseases [2]. Subsequently, the Global Programme to Eliminate Lymphatic Filariasis was established to eliminate LF as a public health problem by the year 2020. The principal strategy used to accomplish this is mass drug administration (MDA), which for most countries means administering a single dose of two drugs (albendazole and diethyl-carbamazine or albendazole and ivermectin) to ≥80% of the entire "at risk" population annually for four to six years [6,7]. Field experience suggests that this strategy will reduce microfilaraemia within the community to very low levels, potentially resulting in permanent interruption of transmission [6]. Follow-up surveys are the principle surveillance mechanism employed to assess the effectiveness of the MDA in decreasing the prevalence of LF in affected populations.

Historically, levels of filariasis and elephantiasis in the Pacific region were some of the highest documented in the world [2]. The Pacific region also has a long history of efforts to control filariasis; however, such efforts have been met with varying success. Some countries such as Wallis and Futuna had persistent and successful elimination programs for decades, whilst many others have experienced resurgence after reducing microfilaria prevalence to levels below 1% [2].

The Pacific Programme to Eliminate Lymphatic Filariasis was launched in 1999, under the auspices of the World Health Organization (WHO). During the past seven years most Pacific Island Countries and Territories (PICTs) have completed initial surveys to map the extent of LF infection. Of the 22 PICTs, 11 were found to be endemic for LF. All endemic countries have commenced MDA with diethyl-carbamazine (DEC) and albendazole, targeting their whole populations, as all people in endemic countries are considered to be "at risk". Five additional countries had evidence of limited focal LF activity and three implemented localised treatment interventions. The principal diagnostic tool used in the Pacific is the immunochromatographic test (ICT), a simple card test with a high reported sensitivity and specificity, that measures the presence of *W. bancrofti *antigen in peripheral blood [8,9].

**Figure 1:**
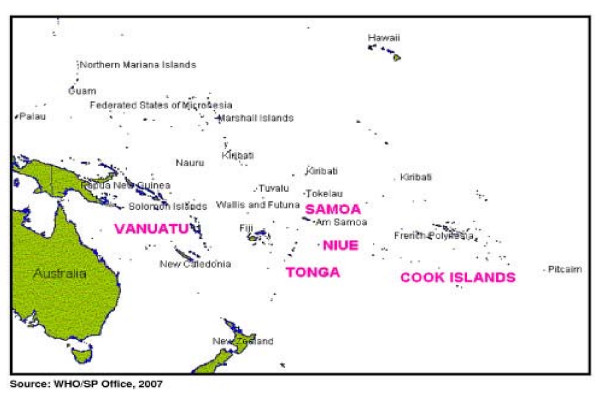
**Map of the Pacific Region, highlighting case study countries**.

By June 2007, five PICTs Cook Islands, Niue, Samoa, Tonga and Vanuatu, had completed five rounds of MDA and a post-MDA survey using a stratified cluster sample design to determine the effectiveness of their MDA program in reaching the goal of <1% antigenaemia prevalence (Figure 1) [2].

The data from these five countries provide an opportunity to reflect on the progress of the Pacific Programme to date and provide information for planning future program activities. Lessons from the Pacific experience highlight some important issues that may be relevant to other LF elimination programs around the world.

## Methods

Data were collected at country level during the delivery of each country's LF elimination program and reported to the WHO to permit collation of country and regional data. Reports included the number of people participating in each round of MDA and the results of the pre- and post-MDA prevalence surveys. It is important to note that regions within countries adopted different approaches to drug distribution using both a central distribution point and less commonly door to door delivery but this was not systematically recorded.

At the commencement of the Pacific Programme to Eliminate Lymphatic Filariasis, each country performed an initial survey to estimate LF antigenaemia prevalence. All countries except Niue reported using *convenience sampling *for these surveys, while Niue tested all residents. Estimates of LF antigenaemia prevalence were made from these surveys using ICT, calculated as the number of ICT positive individuals divided by the number of individuals on whom ICT tests were performed. This was expressed as a percentage and entered onto a spreadsheet, using Microsoft Excel (Microsoft Office XP Professional version). Confidence intervals were not calculated for initial surveys due to the use of convenience sampling.

Each country provided their estimated MDA coverage to the WHO as raw data and percentages of the whole population. This was calculated using the treated population, defined as the number of people provided with medication, divided by the population that was reported by the country's LF program as their total population for each year. This was expressed as a percentage and entered into a Microsoft Excel spreadsheet.

To determine the accuracy of the reported population data for each country, the reported population for each year was compared to other available official sources. To allow comparison across countries, the data for 2001 is presented, the year that three of the five countries conducted a census. Using the population figure supplied by official Government sources from each country, the MDA coverage was recalculated and compared to the reported MDA coverage.

At the completion of five rounds of MDA, four of the case study countries (Cook Islands, Samoa, Tonga and Vanuatu) performed a post-MDA prevalence survey using a *stratified cluster sampling *design to determine if the target of <1% prevalence by ICT had been attained. Stratified cluster sampling was performed by dividing each country into a number of implementation units, with the probability of towns and villages based on population size and geographic location. Within each unit, a random sample of villages was selected and all eligible residents of these villages were encouraged to be tested.

Post-MDA prevalence data was calculated as the number of ICT positive individuals divided by the number of individuals on whom ICT tests were performed. A 95% confidence interval was calculated for all countries other than Niue using the Binomial Stats program, "JavaStat" [10].

Between 1999 and 2004, in contrast to the other countries included in this paper, Niue performed three *whole population *prevalence surveys using ICT. Prevalence was calculated as previously described.

The collection and analysis of data was undertaken as part of routine program activities for the Pacific Programme to Eliminate Lymphatic Filariasis and ethical approval was not required.

## Results

**Figure 2:**
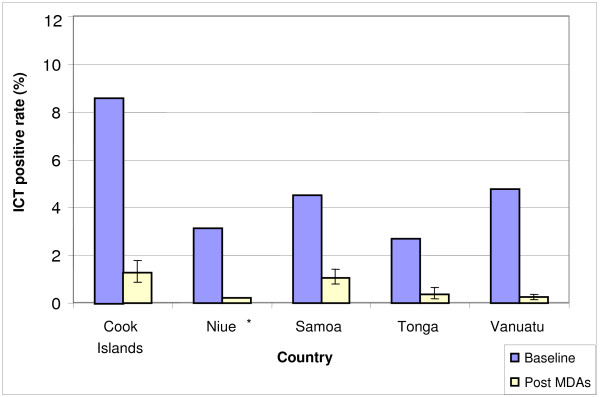
**Lymphatic filariasis antigenaemia prevalence in Pacific case study countries, baseline and post-MDA surveys**.

Initial survey results revealed an ICT prevalence of less than 10% in all five countries, ranging from 8.6% in the Cook Islands to 2.7% in Tonga (Figure 2). The survey methodology was not standardised for Tonga, Samoa, Cook Islands and Vanuatu, and simply described as "*convenience sampling*".

MDA coverage, as reported by each country during the five rounds of MDA varied from 57–99% (Table 1).

**Table 1:** Mass Drug Administration (MDA) coverage reported by country LF programmes

	Reported MDA coverage of population (%)				
COUNTRY	1^st ^MDA	2^nd ^MDA	3^rd ^MDA	4^th ^MDA	5^th ^MDA

Cook Islands	62	64	98	88	93

Niue	94	99	82	78	85

Samoa	90	57	68	60	80

Tonga	79	84	91	86	85

Vanuatu	83	84	84	87	85

For all countries except the Cook Islands, the MDA coverage rate decreased when population totals from official population statistics were used. For example for 2001, a year in which each of the case study countries conducted a census, the comparable MDA coverage using LF program reported population denominators compared to official government statistics, respectively, were: Cook Islands – 64% vs. 77%; Niue – 99% vs. 95%; Samoa – 68% vs. 67%; Tonga – 79% vs. 77%; and Vanuatu – 84% vs. 80%).

The baseline and post-MDA ICT prevalence rates reported by the countries were: Cook Islands 8.6% (162 of 1884 positive) in 1999 to 1.3% (33 of 2202 positive) in 2005; Niue 3.1% (56 of 1794 positive) in 1999 to 0.2% (3 of 1285 positive) in 2004; Samoa 4.5% (317 of 7006 positive) in 1999 to 1.1% (48 of 12719 positive) in 2004; Tonga 2.7% (108 of 4002 positive) in 1999/2000 to 0.4% (11 of 2927 positive) in 2006; and Vanuatu 4.8% (209 of 4362 positive) in 1998 to 0.2% (18 of 7576 positive) in 2005 (Figure 2).

In Niue three cross-sectional population surveys were conducted. Niue reported an initial prevalence of 3.1% by ICT (56/1794). After two and five rounds of MDA, prevalence dropped to 1.5% (22/1630) in 2001 and subsequently to 0.2% (3/1285) in 2004.

## Discussion

Niue provides a unique case study because reliable cross-sectional population data was available. Niue results indicate that five rounds of MDA with coverage between 78–99% can decrease ICT prevalence from 3.1% to 0.2%. This finding supports the premise of the WHO-recommended strategy, that four to six rounds of MDA with high population coverage (≥80%), will successfully reduce the prevalence of LF infections to a level where interruption of transmission is believed to occur [7].

Mathematical modelling by Michael et al (2000) has predicted that post-MDA LF prevalence will be influenced by higher LF prevalence at baseline, high MDA coverage and the use of vector control measures [11]. Unfortunately, inaccurate initial prevalence data preclude exact determination of the change in prevalence for these case study countries, other than Niue. A relatively low initial prevalence (3.1%) and high MDA coverage in Niue (>80% coverage in four MDA rounds) may have contributed to the progress seen in this country.

Niue, Tonga and Vanuatu attained the Pacific Programme's target of an ICT positive prevalence of <1% following five rounds of MDA. The remaining two countries (Cook Islands and Samoa) closely approached but failed to achieve that target. As a result both countries conducted an additional MDA round in 2006, with Cook Islands achieving 94% coverage and Samoa, 93% coverage.

Local vector species and control strategies differ between the case study countries. For these PICTs, the predominant vector species are *Aedes polynesiensis *(Cook Islands, Samoa), *Ae. cooki *(Niue), *Ae. tabu *(Tonga), *Ae. oceanicus *(Samoa, Tonga) and *Anopheles farauti *(Vanuatu) [12]. In addition, Samoa has *Ae. samoanus*, *Ae. tutuilae *and *Ae. upolensis*, while Tonga has *Ae. tongae *[12]. It is of interest that the main LF vector in the two countries with the highest post-MDA survey results (Samoa and Cook Islands) is *Ae. polynesiensis*. This mosquito species is of particular concern in the Pacific region, as there are currently no effective measures of control and it exhibits a trait called "limitation", meaning the mosquito becomes more efficient at transmitting LF when the prevalence within the population is low [12]. The relative contribution of the presence of this vector species, baseline LF antigenaemia prevalence and lower MDA coverage to Samoa and Cook Islands post-MDA prevalence is not known.

Vanuatu's primary LF vector is an *Anopheles sp*., which is also responsible for the transmission of malaria. Vanuatu has been implementing a treated bed-net programme since 1988. Models theorise that adding vector control to MDA campaigns will decrease the number of years required to meet the target prevalence, for a given baseline endemicity [11]. Again, the contribution of an *Anopheles *vector and a treated bed-net programme on the post-MDA prevalence of 0.2% in Vanuatu after five MDAs is not known.

Unfortunately, a lack of data precludes conclusions being drawn about which factors influenced the apparent decrease in prevalence for these countries and the degree to which MDA was associated with a decrease. Most countries used non-standardised methods, with different sample sizes and convenience sampling to establish their prevalence at the start of the programme. While this is understandable due to logistic issues that are common in developing countries [13], it limits comparisons between initial and post-MDA LF antigenaemia prevalence except in Niue where the entire available population was included in each survey.

The Pacific Programme to Eliminate Lymphatic Filariasis' strategy called for five rounds of MDA followed by a prevalence survey to assess the impact. Unlike the global programme, which aimed for ≥80% coverage of the "at risk" population, no specific target was set for the level of coverage to be reached at a national level within each country. Similarly, a coverage survey was not carried out to assess the true coverage achieved. It is likely that higher MDA coverage has contributed to the low prevalence measured in the countries that achieved the programme target. However, it is unfortunate that data limitations preclude exploration of the relationship between coverage levels and program performance. Inaccurate denominator population statistics are common in the Pacific and other regions in the world where LF programmes are underway or planned. Therefore, an independent means of assessing MDA coverage should be considered.

Even if true denominator data was available for each country for every year, estimates of MDA coverage should be viewed with some caution, as it may only reflect drug distribution, rather than drug consumption. A study from India reported that up to 25% of people who received medications during an MDA did not actually take them [14]. Anecdotal reports to the WHO advised that the majority of Pacific MDAs were not "directly observed therapy", and as such, it is unknown whether all drugs were consumed. Directly observed therapy is highly recommended for LF programmes as it may increase compliance with drug administration and allow a more accurate estimate of MDA coverage.

Community members who do not participate in MDA may serve as reservoirs of LF infection [15] and it has been recommended that social research approaches should be used to explore barriers to MDA compliance [16,17]. One of the recommendations of the Pacific Programme to Eliminate Lymphatic Filariasis is that if countries do not reach the post-MDA survey goal of <1% antigenaemia, targeted MDA and vector control should be considered. Clearly administration of targeted MDA will require the identification of groups that do not participate in the MDA and a recommendation of a minimum coverage to be reached. The use of appropriate social science methods in conjunction with a representative MDA coverage survey, could independently measure MDA coverage as well as uncover attitudes, behaviours or beliefs that may impact on the success of future LF elimination efforts. Coverage surveys should also determine what proportion of doses was actually administered under direct observation.

## Conclusion

It is encouraging to observe that countries in the Pacific Programme to Eliminate Lymphatic Filariasis are achieving their LF elimination targets. Niue, Tonga and Vanuatu have all achieved the target of an LF antigenaemia prevalence of <1%, following five rounds of MDA. Although four to six rounds of MDA appear to diminish antigenaemia prevalence, essential for interrupting LF transmission, it is likely that baseline prevalence, MDA coverage and the presence of an efficient vector, such as *Aedes polynesiensis*, are important determinants of post-MDA outcome. This case study from the Pacific highlights the importance of collecting valid and representative data before initiating and during the delivery of public health programmes to learn about the factors that inhibit or promote target attainment.

## Competing interests

The authors declare that they have no competing interests.

## Authors' contributions

CH: analysis and interpretation of data, drafting of manuscript, critical revision of manuscript for important intellectual content. CC: acquisition of data, analysis and interpretation of data, critical revision of manuscript for important intellectual content. KP: acquisition of data, interpretation of data, critical revision of manuscript for important intellectual content. PMK: analysis and interpretation of data, critical revision of manuscript for important intellectual content, study supervision. DND: analysis and interpretation of data, drafting of manuscript, critical revision of manuscript for important intellectual content, study supervision. All authors have read and approved the final manuscript.

## Pre-publication history

The pre-publication history for this paper can be accessed here:

http://www.biomedcentral.com/1471-2334/9/92/prepub
